# New Evidence in Male Infertility Diagnosis: The Role of Metabolomics

**DOI:** 10.3390/cells14231886

**Published:** 2025-11-27

**Authors:** Giuseppina Peluso, Marco Serrao, Lidia Urlandini, Vittoria Rago, Saveria Aquila, Adele Vivacqua

**Affiliations:** 1 Sperm Bank, Department of Maternal Infant, A.O. of Cosenza, 87100 Cosenza, Italy; g.peluso@aocs.it; 2 Department of Urology, Tirrenia Hospital, 87021 Belvedere Marittimo, Italy; marcoserrao81@icloud.com; 3 Department of Pharmacy and Health and Nutrition Sciences, University of Calabria, 87030 Rende, Italy; lidiamaria.urlandini@unical.it (L.U.); vittoria.rago@unical.it (V.R.); 4 Health Center (Cube 34B), University of Calabria, 87030 Rende, Italy

**Keywords:** semen biomarkers, male fertility, metabolomic analysis, sperm quality

## Abstract

**Highlights:**

Unfortunately, male infertility is on the rise worldwide. Sperm quality may influence the outcome of pregnancy in couples. Hence, the identification of new biomarkers may have an important role in the diagnosis and treatment of male infertility. In this context, the omic techniques, particularly metabolomics, can contribute to the early diagnosis of male infertility. This new clinical approach may be considered to improve pregnancy outcomes.

**Abstract:**

Male infertility affects a significant proportion of couples struggling to conceive and re-quire a comprehensive investigation into the underlying causes and potential diagnostic markers. Seminal biomarkers and multi-omic approaches, particularly metabolomics, have provided valuable insights into the alterations of molecular pathways associated with male infertility. This review systematically integrates literature, focusing on studies which examine the interaction between seminal biomarkers, metabolomics, and various omics technologies in the evaluation of sperm quality, and then male infertility. We high-light the importance of a multi-dimensional approach to decipher the complex etiology of male infertility and identify potential diagnostic markers.

## 1. Introduction

**Figure 1:**
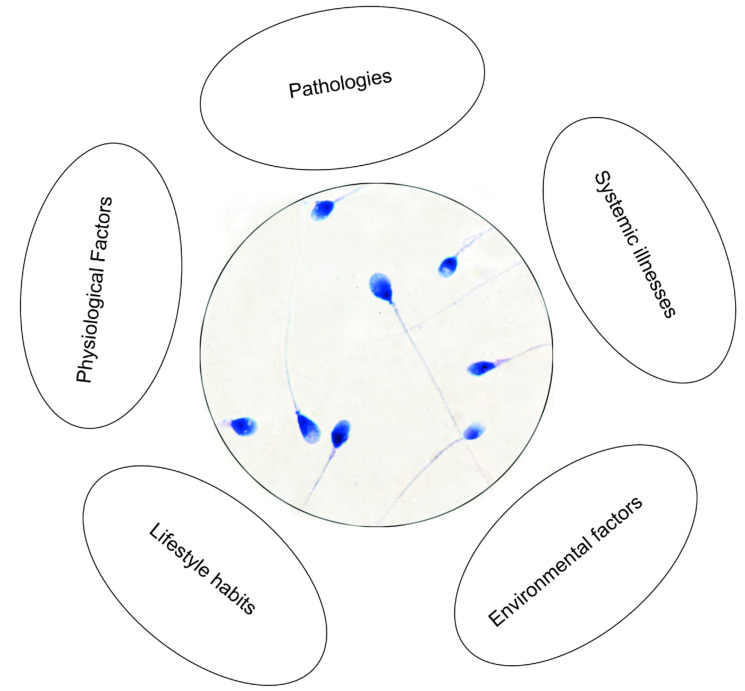
Schematic representation of the main factors involved in men reproductive ability.

Biomarkers are highly sensitive and minimally invasive biological molecules that are used as indicators of body functions. In male infertility, biomarkers aim to assess a man’s potential for fathering children. Currently, semen analysis, by evaluating macroscopic and microscopic factors, is a widely used tool for predicting male fertility potential. While semen analysis provides essential information, significant variability, due to physiological factors, pathologies, systemic illnesses, and environmental factors, such as air pollution, chemicals, and lifestyle habits, there is among the subjects (Figure 1). These variations complicate the interpretation and management of abnormal findings.

An ideal biomarker should detect disease at an early stage, it should be easily measurable, cost-effective, and accurate, having minimal side effects. The discovery of new non-invasive, highly sensitive and specific biomarkers would bring significant benefits to the field of male infertility. These biomarkers could reduce the need for invasive testing in infertile men and enable a more precise and expanded classification of male factor infertility.

The application of novel genetic, proteomic, and metabolomic techniques offers promising avenues for the accurate diagnosis and treatment of male factor infertility [1]. These advanced methodologies provide a foundation for identifying potential biomarkers, which are distinctive biological indicators of processes, events, or conditions that can be objectively measured, evaluated, and compared.

In this context, metabolomics provides a comprehensive overview of the small-molecule metabolites present in biological fluids, reflecting the physiological and pathological state of the organism. Hence, semen metabolomics profiling enables the identification of biomarkers associated with male reproductive function, oxidative stress, and also exposure to environmental contaminants [2,3], contributing to elucidate the complex relationships between semen quality, fertility, and various pathophysiological factors [4].

Typically, metabolomic analyses of seminal plasma are performed using high-resolution analytical techniques such as nuclear magnetic resonance (NMR) spectroscopy, gas chromatography–mass spectrometry (GC-MS), and liquid chromatography–mass spectrometry (LC-MS) [5]. These platforms allow sensitive and quantitative detection of a wide range of metabolites, including amino acids, lipids, and organic acids [6].

Among these techniques, NMR spectroscopy is a non-destructive and very reproducible technique able of providing a wide range of information on many metabolites, using minimal sample preparation. Therefore, NMR is particularly suitable for small volume samples or for preserving biological integrity, however it can identify only compounds present at relatively high concentrations (≥100 µg) [5].

On the contrary, MS, and the more sensitive Ultra-High-Performance Liquid Chromatography coupled with Quadrupole Time-of-Flight Mass Spectrometry (UPLC-QTOF/MS) are techniques, allowing the detection of very low concentration of metabolites (pmol/L to fmol/L) [7].

Hence, the choice of the appropriate metabolomics technique depends on the biological matrix, metabolite characteristics, and research objectives. Table 1 summarizes the main principles, performance features, advantages, disadvantages, and typical applications of the most commonly used metabolomic methods.

Whatever the type of technique used, the data obtained are processed using multivariate statistical analyses (e.g., principal component analysis and partial least squares discriminant analysis) to uncover metabolic patterns linked to specific physiological or pathological conditions [4,8].

For instance, the normal levels of citrate, one of the most established biochemical markers of prostate function, contributing to the regulation of sperm pH, are around 10–50 mmol/L, measurable by High-Performance Liquid Chromatography (HPLC), or Ultra-High-Performance Liquid Chromatography coupled with tandem Mass Spectrometry (UHPLC-MS/MS). Most studies have reported reduced citrate concentrations in men with various semen abnormalities, including azoospermia, oligozoospermia, and idiopathic subfertility, although one study found higher levels in teratozoospermic patients [5]. Lactate, another major metabolite (≈15 mM), also tends to be lower in azoospermic and OAT patients, while some studies show conflicting results in other sub-fertile groups [5]. These discrepancies indicate that the role of lactate in male infertility remains unclear.

Carnitine (L-carnitine and acetyl-L-carnitine), which is associated with sperm motility is detectable in seminal fluid by HPLC, GC-MS, or LC-MS/MS, with typical concentrations of 1–5 mmol/L. Similarly to citric acid, there are no recognised clinical cut-offs. Some studies have shown significant correlations, but clinical validation is still incomplete [9].

The concentrations of glycerol-phosphocholine (GPC) are very variable (0.5–5 mmol/L) and can be detected by ultra-high-performance liquid chromatography with quadrupole time-of-flight mass spectrometry (UPLC-QTOF/MS) or NMR. It is a marker of the epididymis and sperm maturation; however, its clinical use remains experimental, and no standard diagnostic threshold has been defined.

Other metabolites, such as several amino acids (e.g., alanine, arginine, glutamic acid, glutamine, leucine, lysine, phenylalanineproline, tyrosine, valine), spermine, and carnitines, have also shown variable changes between fer-tile and sub-fertile men [10].

However, due to inconsistent findings across studies, further research is needed to clarify their potential as biomarkers of male subfertility Furthermore, metabolomic profiling extends its utility to the enhancement of Assisted Reproductive Techniques (ARTs). Research integrating metabolomic data from semen samples with outcomes of ART has revealed correlations between specific metabolomic profiles and the success rates of these procedures, paving the way for more targeted and effective fertility treatments [11].

The integration of metabolomic methodologies into fertility research represents a significant advance, offering new diagnostic tools and deeper insights into the biochemical underpinnings of male fertility. This approach enhances our understanding of infertility, and improves the predictive power of fertility assessments, ultimately leading to more personalized and effective interventions in reproductive medicine.

In this review, we summarized the current methodologies used to evaluate male fertility, highlighting the importance of metabolomic analysis in the discovering of potential biomarkers useful in preventive and diagnostic approaches.

## 2. Conventional Semen Analysis Versus Metabolomics

The evaluation of semen parameters plays a pivotal role in understanding male fertility potential and diagnosing infertility if associated with an andrological evaluation [11].

The evaluation of male fertility typically involves detailed semen analysis, which is indispensable for diagnosing potential reproductive issues and guiding subsequent medical interventions. Semen analysis is structured into basic examination and extended examination, each targeting different aspects of semen quality to provide a comprehensive assessment. Basic examination encompasses the assessment of macroscopic and microscopic parameters. Macroscopically, it examines the physical characteristics of the semen such as its volume, pH, color, and viscosity. These properties are critical as they provide initial insights into the adequacy of the ejaculate; for example, a low volume or acid or basic pH may indicate problems with seminal vesicles or prostate health, while abnormal color could suggest underlying medical conditions. Viscosity is evaluated to determine the fluidity of semen, with thicker samples possibly impeding sperm movement. Microscopically, this level includes the analysis of sperm concentration, motility, vitality and morphology. Sperm motility is assessed by measuring the percentage of actively moving sperm, crucial for natural conception. Additionally, sperm morphology, which involves the structure and form of spermatozoa, is scrutinized to ensure they are capable of successful egg fertilization. When initial screenings indicate abnormalities or in cases of unexplained infertility, extended examination is employed. This more detailed investigation includes tests such as the detection of anti-sperm antibodies that could be attacking the sperm and impeding their function. Another significant aspect of this level is the evaluation of sperm DNA fragmentation, where the integrity of sperm DNA is measured [12]. High levels of DNA fragmentation are associated with reduced fertility, which might not be detectable through standard semen parameters. Despite their importance in providing a deeper understanding of sperm quality, extended examination tests are not standardized and lack definitive cutoff values, complicating their interpretation. Furthermore, these advanced assessments do not conclusively predict the success of conception, reflecting the complex and multifactorial nature of fertility [12].

Hence, conventional semen analysis provides limited information regarding the molecular mechanisms underlying sperm function and health. With the advent of metabolomics, comprehensive profiling of seminal metabolites provides a novel approach to explore the intricate relationship between metabolism, sperm function, and male reproductive health [13].

Improving male fertility assessment, metabolomics, aids in profiling small metabolites within cells, tissues, or organisms, and has been extensively applied in the analysis of semen to identify biomarkers of sperm health and functionality [13].

This review focuses on recent advancements in semen analysis, through the lens of metabolomics, highlighting its application in areas such as cryopreservation, idiopathic infertility, abortion, and the influence of diet and obesity on semen quality. One of the standouts features to utilize metabolomics in semen analysis is its ability to provide a holistic view of the metabolic profile of an individual’s sperm. Traditional semen analysis methods often fail to reveal the metabolic processes and the impact on sperm quality and functionality. Metabolomics allows for the identification and quantification of a wide range of metabolites within the seminal fluid, providing valuable insights into the metabolic state of the sperm. The integration of multi-omics approaches, including genomics, transcriptomics, and proteomics, has provided a comprehensive view of the molecular mechanisms underlying male infertility. The differential expression of genes and proteins in infertile individuals can unveil potential targets for therapeutic interventions. Moreover, omics-based analysis has shed light on the regulation of sperm metabolism, including glycolysis, Krebs cycle, and oxidative phosphorylation, which play important roles in sperm function and fertility [14].

Metabolomics enables the identification of metabolites that correlate with sperm quality, allowing for accurate diagnosis and prognosis of male infertility. By assessing the levels and types of metabolites present, metabolomics can help detect abnormalities in metabolic pathways that may affect sperm health. Several studies have reported correlations between altered metabolite profiles and impaired semen parameters. For instance, semen samples with reduced sperm motility have shown decreased levels of metabolites involved in energy production and antioxidant defenses, such as Adenosine Triphosphate (ATP), carnitine, and glutathione [15].

Few studies have revealed changes in fatty acid synthesis, ketone body degradation, and lipid metabolism in the spermatozoa and seminal plasma of asthenozoospermic individuals [16,17,18].

Recent research demonstrates the ability of metabolomics to identify specific biomarkers associated with various forms of male infertility. For instance, high-resolution of NMR has been used to detect unique metabolomic signatures in patients with idiopathic oligoasthenoteratozoospermia, which traditional semen analysis often fails to diagnose [19].

The primary application in a real-world clinical setting should entail the ability to pair metabolites commonly measured in biological fluids with others, such that their combined interaction yields distinct outcomes. For example, a serum metabolic profiles study has shown that peptides related to protein complement C3f are potential markers of sperm concentration, opening new avenues for the clinical assessment of male fertility [20]. In particular, C3f peptides reflect local activation of the complement cascade in seminal plasma, as the response to inflammatory or immune processes adversely affecting spermatogenesis. Comparing the seminal plasma peptidome obtained by nanoLC–QTOF-MS from oligozoospermic men respect to normozoospermic subjects, Feng and colleagues identified C3f peptide as significantly correlated with sperm concentration, suggesting that complement activation may contribute to reduced sperm production or survival. However, these findings remain exploratory and require further validation in larger clinical cohorts [21].

Notably, proteomic studies have identified significant proteins that facilitate crucial fertilization processes, such as sperm penetration, the acrosome reaction, and sperm-oocyte fusion. Comparative analyses have highlighted proteins that differ between normozoospermia and conditions like asthenozoospermia, linking these to energy metabolism [22]. These findings suggest that disruptions of the metabolic pathways related to energy metabolism and oxidative stress may contribute to impairing sperm motility [23].

## 3. Metabolomics in Aging

The trend of postponed parenthood has rekindled interest in the age-related decline of testicular function and male fertility. However, the molecular mechanisms underlying testicular aging and the associated decline in fertility remain poorly understood.

Studying the alterations in the metabolomic profile of seminal fluid in correlation with age can be crucial in predicting the health of the offspring [24]. The effects of aging on metabolite secretion and DNA integrity cannot be overlooked. To mitigate these issues, which often coincide with advanced maternal age, metabolomic analysis can provide significant advantages [25].

The aging process is marked by a gradual deterioration of various organic, physiological, and metabolic functions, the mechanisms of which have largely remained elusive, and metabolomics has emerged as a pivotal tool in this exploration. Through the identification of specific metabolites and their fluctuations, metabolomics has the potential to uncover the biochemical activities that characterize aging. This includes the unraveling of pathways involved in energy metabolism, cellular repair, oxidative stress, and inflammation, among others. Understanding these pathways helps to clarify how physiological and metabolic functions deteriorate over time and may also illuminate strategies to mitigate these effects, thereby enhancing health span.

A retrospective analysis conducted on 12,828 sperm samples from aged and young male groups, each consisting of thirty participants divided by age, UPLC-Q-TOF/MS identified 129 differentially expressed metabolites, 62 downregulated and 67 upregulated, between the groups. These metabolites were significantly enriched in key metabolic pathways such as the citrate cycle (TCA cycle), Parkinson’s disease, cholesterol metabolism, and glycerophospholipid metabolism, as indicated by Kyoto Encyclopedia of Genes and Genomes (KEGG) pathway analysis [26]. The study also observed a positive correlation between male age and sperm DNA fragmentation index (DFI), and a negative correlation with semen volume and motility, aligning with previous studies suggesting that age impacts sperm concentration and fertilization capacity. A decrease in semen volume likely results from the diminished function of accessory glands in the aging male reproductive tract, leading to an increase in sperm concentration when total sperm count remains unchanged. The analysis underscored the role of oxidative stress in aging semen, with a focus on the balance of glutathione metabolism. Reduced levels of citrate and key metabolites in seminal plasma were associated with impaired sperm function, while elevated levels of biomarkers, like carnitine suggested disrupted fatty acid metabolism. This study also established a biomarker panel using an integrated machine-learning approach, identifying metabolites strongly correlated with aging semen, indicative of reduced sperm functionality [26]. In a recent study, Castro analyzed 138 samples from apparently healthy untrained individuals [25]. They found a positive correlation between age and several clinical markers, including total cholesterol, High-Density Lipoprotein (HDL), Low-Density Lipoprotein (LDL), Very Low-Density Lipoprotein (VLDL), triacylglyceride, and glucose levels. Conversely, age showed a negative correlation with serum levels of Branched-Chain Amino Acids (BCAAs) such as tryptophan, 3-hydroxyisobutyrate, asparagine, isoleucine, leucine, and valine. Interestingly, there was a positive correlation between age and levels of aspartate and ornithine. The metabolites correlating with age, were linked to three key metabolic pathways: the degradation of valine, leucine, and isoleucine; the urea cycle; and ammonia recycling. The data suggest that aging is associated with reduced levels of BCAAs, particularly noticeable after the third decade of life. This reduction could reflect changes in muscle mass and function, as BCAAs are crucial for muscle protein synthesis and maintenance. Additionally, increased levels of metabolites related to the urea cycle in older individuals contribute to enhance protein breakdown and ammonia detoxification with age. The study showed the decreasing of serum metabolic levels of specific metabolites related to valine, leucine, and isoleucine degradation pathways and the increasing of aspartate, and ornithine, indicating a significant metabolic shift related to aging [25]. Furthermore, several researchers have also focused on animal studies, to investigate the relationship between the metabolic and metabolomic profiles and testicular tissue. Using NMR spectroscopy, a sophisticated tool for determining the composition of biological samples, they studied the biochemical changes occurring within the tests of aged versus younger rats. The analysis revealed significant alterations in the metabolic pathways related to phospholipid metabolism in the testicular tissue of aged rats. Specifically, there was a notable increase in the precursors of phospholipids coupled with a decrease in their phosphorylated end products. This imbalance suggests a disruption in phospholipid synthesis, which is crucial for maintaining cell membrane integrity and signaling pathways essential for spermatogenesis. Phospholipids and their derivatives play pivotal roles in cell membrane composition and function, and their disrupted balance could impact on the spermatogenic process. This alteration in the metabolic landscape could potentially lead to reduced fertility or other reproductive deficiencies associated with aging [25,27].

## 4. Metabolomics in Sperm Cryopreservation

The cryopreservation of semen is an invaluable tool for the preservation of genetic resources across a wide range of species, enabling the conservation and reconstruction of populations and genetic diversity. In humans, cryopreservation of seminal fluid samples is currently a valuable resource, both in the context of onco-fertility and fertility preservation, as well as for the possibility of storing samples to plan pregnancies or align with the timing of ART. Despite advancements in freezing techniques aimed to preserve the health and integrity of spermatozoa [28].

The process of cryopreservation in liquid nitrogen inevitably induces changes in the metabolic profile of gametes and can also affect the quality of seminal plasma. Investigating metabolic and genetic differences among individuals might offer a personalized-based direction for advancing semen cryopreservation research. The primary goal of cryopreserving spermatozoa and spermatogenic cells is to ensure the delivery of an intact genome, presenting a distinct set of technical challenges. Over the past few decades, semen cryopreservation research has been predominantly empirical, focusing on varying technical parameters such as cryoprotectant concentrations, additives, and cooling and warming rates. While these studies may enhance post-thaw sperm quality under specific conditions, they do not identify specific molecular profiles that can be important in predicting the freezing–thawing cycle success.

Recognizing these limitations, some research groups have shifted their focus towards modeling the hydraulic fluxes, i.e., the changes in osmotic pressure that cause intra and extracellular water movement during the freezing–thawing process of spermatozoa [28]. This approach aims to provide a more comprehensive understanding of the factors influencing sperm survival and functionality post-cryopreservation, moving beyond empirical studies to establish a more robust scientific foundation for improving cryopreservation techniques.

Metabolomics has identified biomarkers predictive of cryopreservation outcomes, such as changes in lipid metabolism and energy pathways which correlate with sperm viability post-thaw [28]. Currently, in the literature there are several studies conducted on the seminal fluids of various animal species (boar, horse, goat, ram, turkey, etc.) to evaluate the impact of cryopreservation on the metabolomic profile [29,30,31,32,33,34]. Seminal plasma, composed of secretions from the testis, epididymis, and accessory sex glands, plays a vital role in modulating sperm function. While some potential markers of boar sperm freezability have been identified in spermatozoa, less attention has been given to seminal plasma. The exposure of spermatozoa to metabolites in seminal plasma can significantly affect their function, the detailed significance of the seminal plasma metabolome related to sperm freezability remains unknown yet. Addressing this gap, recent studies have utilized ultra HPLC (UHPLC) to restore the component and the Q-TOF-MS to identify and quantify it. UHPLC-qTOF-MS analysis to identify 953 metabolites in boar seminal plasma, finding 50 metabolites with significant differences between Good Freezability (GFE) and Poor Freezability (PFE) groups. Further, metabolic target analysis identified three key metabolites—D-aspartic acid, N-acetyl-L-glutamate (NAG), and inosine exhibited significant differences. These metabolites may serve as potential markers for assessing sperm cryopreservation resistance in boars [29].

Seminal plasma contains a variety of organic and inorganic components essential for sperm function, including amino acids, proteins, ions, sugars, and lipids. These components provide energy and serve as a transport medium for sperm. Seminal plasma plays a crucial role in regulating key biological processes in sperm, such as hyperactivation motility, the acrosome reaction, and capacitation. However, its role in semen cryopreservation is challenging to ascertain due to the complexity of its components; in fact, conflicting results have emerged regarding the effects of seminal plasma on sperm function post-cryopreservation.

For example, Moore found no significant effect on sperm motility in equine spermatozoa exposed to seminal plasma after cryopreservation, though prolonged exposure before freezing was detrimental [30]. Conversely, seminal plasma improved the progressive motility and structural integrity of boar sperm during cryopreservation [29]. These variations in function could be due to differences in the composition of seminal plasma, influenced by the anatomical structure of accessory sex glands.

Notably, differences in the contribution of seminal plasma to sperm cryopreservation have been observed even within the same species. For instance, sperm from low-resilience rams exhibited higher viability when frozen with seminal plasma from high-resilience rams [32]. Similarly, in stallions, supplementation with high cryo-resistance seminal plasma improved sperm fertility and zona pellucida binding [30]. These findings highlight the significant variation in small molecular metabolites within seminal plasma, emphasizing its critical role in sperm cryopreservation.

Another model, studied to evaluate variations in the metabolomic profile of seminal fluid is the turkey as the freezing–thawing process significantly reduces sperm quality, primarily due to an insufficient understanding of the molecular mechanisms underlying cryopreservation. Using NMR technology to assess metabolic changes in turkey sperm during cryopreservation and correlating these changes with sperm quality, researchers identified metabolic markers related to sperm freezability. L-glutamine, L-aspartate and L-arginine were directly involved in sperm freezability whereas other molecules such as ketoisocaproic acid, choline and benzoic acid are involved by participating in anabolic processes important in sperm viability [33]. This may suggest dietary or medium supplementation strategies to enhance cryopreservation outcomes.

In addition, significant differences have been observed between the metabolites in freshly analyzed samples and those analyzed after cryopreservation. Using LC-MS, samples from high- and low-fertility bulls were examined. Cryopreservation increased lysophosphatidylcholine in seminal plasma and elevated glycine betaine and pyro-L-glutaminyl-L-glutamine levels in spermatozoa. High-fertility bulls had higher levels of L-acetylcarnitine, glycerol tripropanoate, 2,3-diacetoxypropyl stearate, and glycerophosphocholine, but lower levels of lysophosphatidylcholine and butyrylcarnitine in fresh seminal plasma compared to low-fertility bulls. Additionally, fresh spermatozoa from high-fertility bulls showed more glycerophosphocholine and lysophosphatidylcholine. In cryopreserved samples, high-fertility bulls exhibited increased glycerophosphocholine and decreased butyrylcarnitine, glycine betaine, and L-carnitine in seminal plasma, along with lower glycine betaine in spermatozoa. These findings highlight that cryopreservation affects the metabolomes of both seminal plasma and spermatozoa, with distinct differences between high- and low-fertility bulls [34].

In conclusion, while empirical studies have contributed to increment sperm cryopreservation, a shift towards genomics-based research and biophysical modeling may offer new insights and strategies for enhancing sperm survival and quality. By addressing the genetic and biophysical underpinnings of sperm cryopreservation, future research can develop to be more effective and predictive methods for preserving male fertility across various species.

## 5. Unexplained Male Infertility (UMI)

One of the primary applications of metabolomics, alongside proteomics and genomics, is the search for potential biomarkers related to infertility, particularly unexplained infertility. Among infertile couples, approximately 50% of cases are attributed to male factors. Of these, around 18% experience idiopathic infertility, where the underlying cause remains unknown [35]. The identification of biomarkers in this context is crucial for improving diagnosis and treatment strategies, offering hope for more personalized and effective approaches to managing male infertility. The biomarkers identified through metabolomics, may be related to the pathogenesis of UMI, offering novel therapeutic targets.

Seminal plasma has long been a key source to investigate male infertility. Studies have shown that the metabolomic profiles of infertile patients differ significantly from those of fertile men. Metabolic fingerprinting of seminal plasma has been proposed as a screening tool for male infertility. While genomic and proteomic approaches have contributed to understanding male infertility, metabolomics is emerging as a powerful technology for diagnosing diseases and uncovering mechanisms linked to disease processes. Metabolomic analysis of serum or urine samples has shown potential in male infertility research, but changes in the seminal plasma metabolome, which directly reflects the chemical environment and function of sperm, remain unclear [36].

To deeper understand these mechanisms, Qiao used a GC-MS-based metabolomics approach, profiled the metabolites in the seminal plasma of UMI patients [37]. They identified 153 metabolites distinguishing 82% of UMI patients from healthy controls with 92% specificity. Among these metabolites, 44 were differentially expressed in UMI subject. This is an important result underlying that metabolomics, especially in cases of idiopathic infertility, offers a window into unexplained etiological factors. Furthermore, alterations in semen metabolites related to carbohydrate metabolism and oxidative stress have been linked with idiopathic non-obstructive azoospermia, suggesting potential metabolic dysregulations impacting sperm production and quality.

## 6. Abortion and Semen Quality

Metabolomic profiling has recently been implicated in elucidating male factors associated with unexplained Recurrent Spontaneous Abortion (RSA), suggesting that semen quality substantially influences reproductive success and outcomes [38]. RSA, characterized by two or more consecutive pregnancy losses before 20 weeks of gestation, affects approximately 1–2% of couples attempting to conceive and presents a significant challenge in reproductive medicine. Currently, the etiology of RSA remains undetermined in up to 50% of cases, classified as Unexplained RSA (URSA) [39].

Historically, investigations into RSA have predominantly focused on female factors. However, considering that male gametes contribute 50% of the genomic material to the zygote, their role in successful embryonic development and live births, has been estimated to influence 10–15% of outcomes [39]. URSA represents a particularly perplexing outcome within this context, with current evidence often limited by small cohort size [39]. The integration of metabolomic data in this field could therefore provide critical insights into male contributions to RSA, potentially leading to more targeted interventions, improving reproductive outcomes.

The importance of sperm morphology in this context has long been emphasized. Indeed, while a metabolic profile directly reflects cellular metabolic functionality, it is also true that morphology inherently, serves as a predictive value for cellular functionality and fertilizing capacity. Specifically, it has been shown that men linked to RSA exhibit a significantly lower proportion of normally shaped sperm and a higher incidence of morphological abnormalities [40]. Additionally, it is important to consider that one of the reasons for the increased risk of RSA is the advancing age of women and men, causing in this later the altered sperm morphology, as mentioned above. As demonstrated by Zhang and colleagues, spermatozoa are the target cells of several metabolites, and seminal plasma provides a nourishing and protective medium for spermatozoa during their journey through the female reproductive tract [38]. Therefore, the measurement of biomarkers in spermatozoa and seminal plasma represents a non-invasive indicator for the clinical detection of abnormalities. This study demonstrated that reduced sperm count, decreased motility, and a lower percentage of normal sperm, together with metabolic alterations, are associated with URSA. Potential biomarkers of ascorbic acid and guanine in seminal plasma and hexadecanedioic acid and pyroglutamic acid in spermatozoa, along with key metabolic changes, including oxidative stress and hormonal metabolism in spermatozoa, nucleic acid synthesis and oxidative stress in seminal plasma, provide a new scientific basis for the prediction and treatment of URSA from the perspective of seminal factors.

## 7. Lifestyle

The impact of lifestyle on sperm quality covers multiple factors, including diet, environmental exposure, and psychological stress. Among these, diet, as a modifiable factor, has attracted significant attention for its role in influencing sperm quality through metabolomics. In particular, sperm motility, closely associated with pregnancy success, is frequently abnormal in obese men, with a significant prevalence observed in studies. Optimizing body mass index (BMI) in obese individuals has been shown to improve sex hormone levels, erectile function, and seminal parameters, indicating a potential reversibility of these effects [41]. Although numerous meta-analyses and cross-sectional studies have shown discrepancies in correlating semen quality with BMI [42,43], it becomes clear that metabolomic analysis of semen can provide meaningful data to determine an individual’s fertility potential and health status. Biomarkers potentially identified with metabolomic approaches help understand how factors such as nutrition and BMI influence semen quality. For example, antioxidant-rich diets have been shown to improve semen quality by reducing oxidative stress, a well-known factor in the etiology of semen disorders [43], while a high intake of red and processed meats, saturated fats, and low intake of fruits and vegetables have been linked to reduced sperm parameters [44]. Obesity alters the nature of metabolic signatures in semen, influencing sperm motility and concentration, with specific fatty acids and amino acids playing a critical role [43]. In addition, higher BMI levels are associated with hormonal imbalances that may negatively impact sperm quality. In this context, studies using LC–MS, GC–MS, and NMR spectroscopy have revealed that semen from obese men exhibits a distinct metabolic signature characterized by elevated levels of leucine, isoleucine and valine, reduced carnitine and antioxidant metabolites, altered lipids and increased oxidative stress markers [45,46,47]. For instance, the elevated levels of these BCAAs may activate mTORC1 signaling, shifting the energy balance toward anabolic processes and suppressing autophagy. In spermatozoa, such metabolic change leads to decreased ATP availability for flagellar movement and increased production of ROS due to incomplete mitochondrial oxidation. In turn, ROS accumulation promotes oxidative modifications of axonemal and mitochondrial proteins, thereby impairing motility [48]. Theses alterations are representative of metabolic diseases such as insulin resistance, dyslipidemia, and chronic inflammation, which in turn may influence sperm metabolism and motility [49].

Obesity-associated dyslipidemia alters both systemic and seminal lipid profiles, leading to increased levels of saturated and oxidized lipids, ceramides, and diacylglycerols (DAGs) [50]. These lipids activate protein kinase C (PKC) and JNK stress pathways, promoting apoptosis, mitochondrial dysfunction, and loss of motility. Furthermore, peroxisome proliferator-activated receptors (PPARs), particularly PPARα and PPARγ, regulate lipid utilization, mitochondrial biogenesis, and antioxidant defense. In obesity, downregulation of PPARs signaling has been observed in testicular and sperm cells, exacerbating lipid peroxidation and compromising membrane integrity and motility [51].

The metabolic derangements described above converge on mitochondrial dysfunction, the principal cause of reduced sperm motility. In obese males, seminal plasma exhibits decreased antioxidant capacity (e.g., reduced glutathione, catalase, and superoxide dismutase activity) and increased oxidative markers (malondialdehyde, 4-hydroxynonenal) [52].

Excess ROS directly oxidize PUFA residues in sperm membranes, reducing fluidity and damaging axonemal components critical for flagellar motion. Moreover, mitochondrial ROS induce mtDNA damage, depolarization of the inner mitochondrial membrane, and decreased oxidative phosphorylation efficiency, all of which impair the energy supply for motility [46,52].

Studies have indicated that epigenetic regulation may play a pivotal role in male fertility by orchestrating gene expression during spermatogenesis, sperm maturation, and early embryonic development. Alterations in DNA methylation, histone modifications, and small non-coding RNA profiles can compromise sperm function and the ability to transfer a correct epigenetic information to the embryo [53,54,55].

Such changes can impair chromatin condensation, reduce sperm motility, and alter fertilization competence. Dysregulation of specific sperm microRNAs, for instance miR-34c and miR-15b, has been linked to defective mitochondrial function and abnormal apoptosis control, further compromising sperm viability [56].

Of note, these epigenetic alterations are not merely markers of sperm dysfunction but may also mediate intergenerational inheritance of metabolic disorders. Evidence from both human and animal studies indicates that paternal obesity and metabolic syndrome can modify sperm DNA methylation patterns and miRNA content, transmitting altered metabolic traits and developmental risks to the offspring [57,58].

Thus, the crosstalk between metabolic imbalance and epigenetic remodeling provides a critical mechanistic link between environmental exposures, sperm function, and male fertility outcomes. Table 2 reports the main molecular mechanisms linking obesity and sperm quality.

While seminal fluid analysis can already assess some lifestyle-induced alterations, it is often insufficient. Stress factors—whether mechanical, emotional, environmental pollutants, or conditions such as sports or occupations that increase scrotal temperature, can significantly disrupt seminal fluid quality. These changes correlate with alterations in an individual’s metabolomic profile and can provide a substantial advantage in evaluating and predicting an individual’s fertility potential. This is especially true when combined with metabolomic profiling and the assessment of environmental contaminants, enhancing the predictive accuracy of fertility evaluations. Endocrine disrupting chemicals (EDCs) are a significant global public health concern; it has been recognized to interfere with hormone action. Common EDCs include bisphenol A (BPA), phthalates, parabens, triclosan (TCS), and UV light filters, which are prevalent in a variety of consumer products ranging from plastic bottles and cosmetics to personal care products and kitchenware. Despite the known presence of EDCs in the environment and their detection in human urine, only a limited number of studies have measured these chemicals in seminal fluid and assessed their association with semen quality [59,60,61]. This gap in research highlights the need for further investigation into the impact of EDCs on male reproductive health, using seminal plasma to better understand the relationship between EDC exposure and semen quality parameters. The environmental presence of EDCs is of particular concern due to their potential impact on male reproductive health [59,60]. Research indicates that many EDCs can generate reactive oxygen species, leading to oxidative stress that may result in sperm DNA damage, reduced sperm count, and poor sperm motility and morphology [62]. Seminal plasma, containing secretions from male accessory sex glands crucial for sperm maturation and conception, has been suggested as a potential marker of male fertility and a proxy to assess male target tissue dose of EDCs [62].

## 8. Discussion

Advancements in non-invasive metabolomic techniques and high-throughput multi-omics platforms offer opportunities for large-scale studies and the discovery of novel biomarkers. However, challenges such as standardization of protocols, sample size limitations, and data integration remain to be addressed for the widespread clinical implementation of these approaches. Continued research and collaboration with different disciplines are crucial to further unravel the intricate mechanisms underlying male infertility and translate these findings into clinical practice.

In seminal research, metabolomics has been applied to decipher the complex metabolic interactions that contribute to male infertility. Studies employing high-resolution NMR spectroscopy and mass spectrometry have revealed distinctive metabolic profiles in the seminal plasma of men with idiopathic infertility [13]. These profiles include alterations in metabolites related to oxidative stress, sperm energy metabolism, and cell membrane functionality [2,3]. One pivotal study demonstrated that metabolomic profiling could effectively differentiate men with idiopathic infertility from healthy controls, identifying specific biomarkers such as citrate, lactate, and various amino acids that are significantly altered in infertile patients. These biomarkers are linked to energy production and antioxidant defense mechanisms crucial for maintaining sperm viability and motility [63]. Another research has focused on the predictive potential of these metabolic alterations in clinical outcomes of assisted reproductive technologies. For instance, metabolic shifts in seminal plasma are associated with the fertilization potential of spermatozoa and embryo quality, providing insights that could guide therapeutic interventions [64]. A comprehensive systematic review highlighted the relevance of metabolomics in reproductive medicine, suggesting that metabolic profiling could serve as a non-invasive diagnostic tool to improve the management of male infertility [44]. These studies indicate that metabolomics can aid to elucidate the etiological factors of infertility, providing a foundation for personalized medicine approaches in andrology.

One of the pioneering studies employed NMR technology to discern metabolic changes in seminal plasma across different groups, including those with vasectomy, severe oligoasthenozoospermia, and normozoospermia [65]. Notable, findings included significant alterations in glycerylphosphorylcholine, citrate, and lactate levels in the azoospermia group compared to normozoospermic subject [65].

For patients with spinal cord injuries, significant dysregulation in glycerolipids and glycerophospholipids was found [66]. Similarly, comparing metabolomic profiles across various infertility groups, key metabolites like arginine, citrate, and fructose were identified to be differentially regulated.

Recent advancements have utilized non-targeted metabolomics profiling to explore broader metabolic changes in conditions like asthenozoospermia and Kidney-Yang deficiency syndrome (KYDS) in infertile males. For instance, it was identified 19 deregulated metabolites in asthenozoospermia patients, indicating disruptions in lipid metabolism and the Krebs cycle, among others, suggesting a metabolic basis for this syndrome [67].

Furthermore, the potential for metabolomics to aid in noninvasive diagnostics has been shown in studies analyzing non-obstructive azoospermia. Techniques like metabolic fingerprinting have been proposed as rapid diagnostic tools to detect spermatogenesis issues in such cases to identify metabolic conditions potentially related to non-obstructive azoospermia.

Lastly, recent research in urinary and plasma metabolomics has sought to identify potential biomarkers for male infertility, with studies finding associations between specific metabolites and semen quality.

## 9. Limitations of Current Research

Current metabolomics research in male infertility has limitations due to small sample sizes, poor validation, and the need for integrated approaches that combine metabolomics with other “omics” technologies. Indeed, many studies fail to adequately explain the complex correlations between metabolic changes and specific sperm alterations [68]. Furthermore, for clinical application of metabolomics, it must be integrated with other fields such as genomics and proteomics. This integration is made difficult by the lack of experts, the need for further research, and the costs and regulatory approval processes that represent the main obstacles to integrating these technologies into clinical practice.

Future research should focus on standardizing metabolic biomarkers for clinical use, integrating metabolomic data with other “omics” to improve precision diagnostics, and applying metabolomics to understand the molecular basis of male infertility. Further studies could focus on developing targeted and personalized treatments based on the role of the seminal microbiome and environmental factors.

## 10. Conclusions

**Figure 2:**
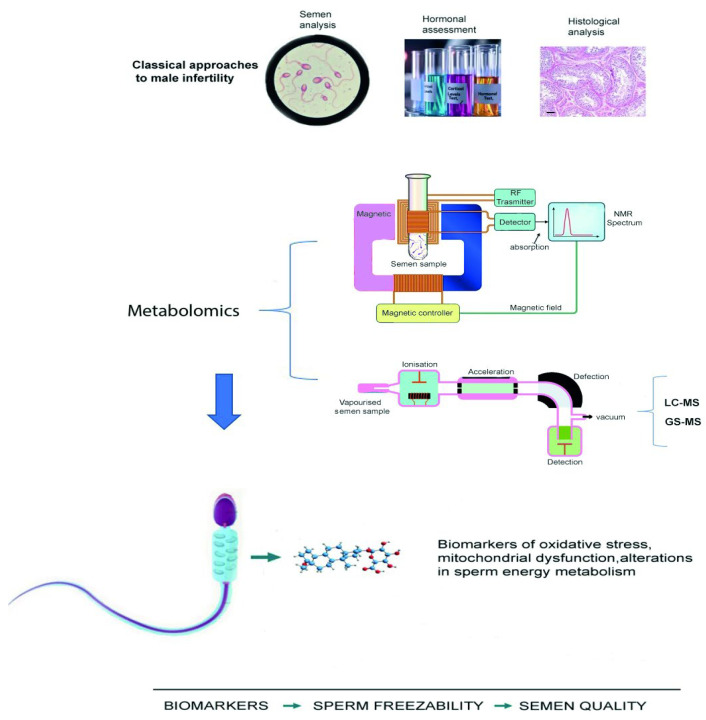
Schematic overview of classical and metabolomics-based approaches in the evaluation of male infertility. The scale bar of the figure in 25 µm.

In conclusion, the application of metabolomics in the field of male idiopathic infertility is proving to be a crucial advancement, offering novel insights and potential biomarkers for diagnosing and treating this complex condition (Figure 2).

The integration of metabolomic data with clinical practices promises to enhance the understanding and management of male infertility, paving the way for tailored therapeutic strategies that address the unique metabolic dysfunctions of affected individuals. This highlights the growing importance of metabolomics in understanding and diagnosing male reproductive problems, although the field continues to evolve with the need for ongoing research to further clarify these metabolic interactions.

## Figures and Tables

**Table 1:** Comparison of metabolomics technical methods.

Technical Method	Principle	Resolution	Detection Time	Advantages	Disadvantages	Application
**NMR**	Based on nuclear spin magnetic resonance properties in a magnetic field	Medium	Short	Non-destructive, highly reproducible, minimal sample preparation, no derivatization required	Low sensitivity, limited dynamic range	Rapid profiling of metabolic patterns in biological fluids such as urine, plasma, semen
**UPLC-QTOF/MS**	Combines high-efficiency chromatographic separation with high-resolution mass spectrometric detection	High	Moderate	High sensitivity and resolution, wide metabolite coverage, accurate mass de-termination	Requires sample pretreatment, high operational cost, potential ion suppression	Detection of trace metabolites such as hormones, lipids, and small pep-tides
**GS-MS**	Separates volatile compounds by gas chromatography followed by mass spectrometric detection	High	Moderate to long	Excellent separation of volatile and thermally stable com-pounds, established compound libraries	Requires derivatization of non-volatile compounds, destructive analysis	Analysis of volatile or semi-volatile metabolites such as fatty acids, amino acids, organic acids

NMR = Nuclear Magnetic Resonance; UPLC-QTOF/MS = Ultra-Performance Liquid Chromatography–Quadrupole Time-of-Flight Mass Spectrometry; GC-MS Gas = Chromatography–Mass Spectrometry.

**Table 2:** Key Molecular Mechanisms Linking Obesity to Altered Semen Metabolism.

Conditions	Molecular Pathway	Effect on Sperm
**Insulin resistance**	Impaired glucose and amino acid metabolism in Sertoli and germ cells	Reduced energy substrate supply
**Oxidative stress**	Overproduction of ROS, reduced antioxidant enzymes	Lipid peroxidation, DNA damage, reduced motility
**Mitochondrial dysfunction**	Decreased ATP synthesis, depolarized membrane potential	Flagellar motion impairment
**Inflammation**	Cytokine signaling affects testicular metabolism	Spermatogenesis defects
**Epigenetic alterations**	DNA methylation and microRNA expression changes	Affects genes controlling energy metabolism and motility

## Data Availability

No new data were created or analyzed in this study.
